# Cost Resulting from Anti-Tuberculosis Drug Shortages in the United States: A Hypothetical Cohort Study

**DOI:** 10.1371/journal.pone.0134597

**Published:** 2015-08-18

**Authors:** James C. Scott, Neha Shah, Travis Porco, Jennifer Flood

**Affiliations:** 1 Colby College, Department of Mathematics and Statistics, Waterville, Maine, United States of America; 2 Francis I. Proctor Foundation, San Francisco, California, United States of America; 3 California Department of Health Tuberculosis Control Branch, Richmond, California, United States of America; 4 Centers for Disease Control and Prevention, Division of Tuberculosis Elimination, Atlanta, Georgia, United States of America; 5 Department of Epidemiology and Biostatistics, University of California San Francisco, San Francisco, California, United States of America; Indian Institute of Science, INDIA

## Abstract

**Background:**

From 2012 through 2014, the United States experienced acute shortages and price escalations of several first-line anti-tuberculosis (TB) medications. Because secondary TB drug regimens are longer and adverse events occur more frequently with them, we sought to conservatively estimate the cost, to patients and the health care system, of TB treatment and medication adverse events from alternative regimens during drug shortages.

**Methods:**

We assessed the cost of treatment for TB disease in the absence of isoniazid (INH), rifampin (RIF), or pyrazinamide (PZA), or both INH and RIF. We simulated adverse events based on published probabilities using a monthly discrete-time stochastic model. For total costs, we summed costs of medications, routine testing, and treatment of adverse events using procedural terminology codes. We report average cost ratios of TB treatment during drug shortages to standard TB treatment.

**Results:**

The cost ratio of TB treatment without INH, RIF, or PZA to standard treatment was 1.7 (Range: 1.2, 2.3), 4.9 (Range: 3.2, 7.3), and 1.1 (Range: 0.7, 1.7) times higher, respectively. Without both INH and RIF, the cost ratio was 18.6 (Range: 10.0, 39.0) times higher. When the prices for INH, RIF and PZA were increased, the cost for standard treatment increased by a factor of 2.7 (Range: 1.9, 3.0). The percentage of patients experiencing at least one adverse event while taking standard therapy was 3.9% (Range: 1.3%, 11.8%). This percentage increased to 51.5% (Range: 20.1%, 83.8%) when RIF was unavailable, and increased to 82.5% (Range: 41.2%, 98.5%) when both INH and RIF were unavailable.

**Conclusions:**

Our conservative model illustrates that an interruption in first-line anti-TB medications leads to appreciable additional costs and adverse events for patients. The availability of these drugs in the United States should be ensured. Models that incorporate the effectiveness of alternative regimens, delays in treatment initiation, and TB transmission can provide broader perspectives on the impact of drug shortages.

## Introduction

Tuberculosis (TB) results from infection with the bacterium *Mycobacterium tuberculosis*. It spreads from person to person primarily through the air, and it most often affects the lungs. If not treated promptly, it can be fatal. The Centers for Disease Control and Prevention (CDC) reported 9,582 new cases of TB in the United States in 2013, representing a 3.6% decline from 2012 [1]. In most instances, TB can be cured. First-line treatment consists of four medications—isoniazid (INH), rifampin (RIF), ethambutol, (EMB), and pyrazinamide (PZA)—taken for 6–9 months. However, drug resistant strains of TB are emerging, and these may require longer treatment with second-line medications that often are more toxic and require longer terms of treatment. Halting TB transmission and decreasing morbidity and mortality requires prompt diagnosis and initiation of effective therapy.

In November 2012, CDC reported an interruption in the supply of INH [2, 3]. In addition to being a first-line medication for the treatment for TB disease, INH alone or in combination is one medication recommended for treatment for latent *Mycobacterium tuberculosis* infection (LTBI) to prevent progression to TB disease. The supply interruption continued for several months, and reports of procurement challenges persist [4]. Because of this interruption, TB program officials were forced to reorder their priorities for preventive treatment and to change to alternative and, in some instances, more expensive treatment regimens for both latent infections and TB disease [5]. Unfortunately, supplies of other anti-TB medications besides INH have been interrupted, as well. Since 2005, CDC has tracked shortages of second-line anti-TB medications including streptomycin, cycloserine, ethionamide, rifabutin, amikacin, capreomycin, and kanamycin.[3]. Furthermore, in 2014, the National Tuberculosis Controller’s Association (NTCA) reported substantial cost escalations in three of the four first-line anti-TB medications: INH, RIF, and PZA[4, 6]. Shortages of anti-TB drugs and costs increases can impede progress toward the ultimate goal of TB elimination.

A better understanding of the impact of anti-TB medication shortages is needed to guide future national and local TB control strategies, including medication procurement policies and resource allocation. The goal of our investigation was to examine the financial impact on the TB program resulting from TB medication shortages and price fluctuations of INH, RIF and PZA, on patients and health care systems.

## Methods

**Fig 1:**
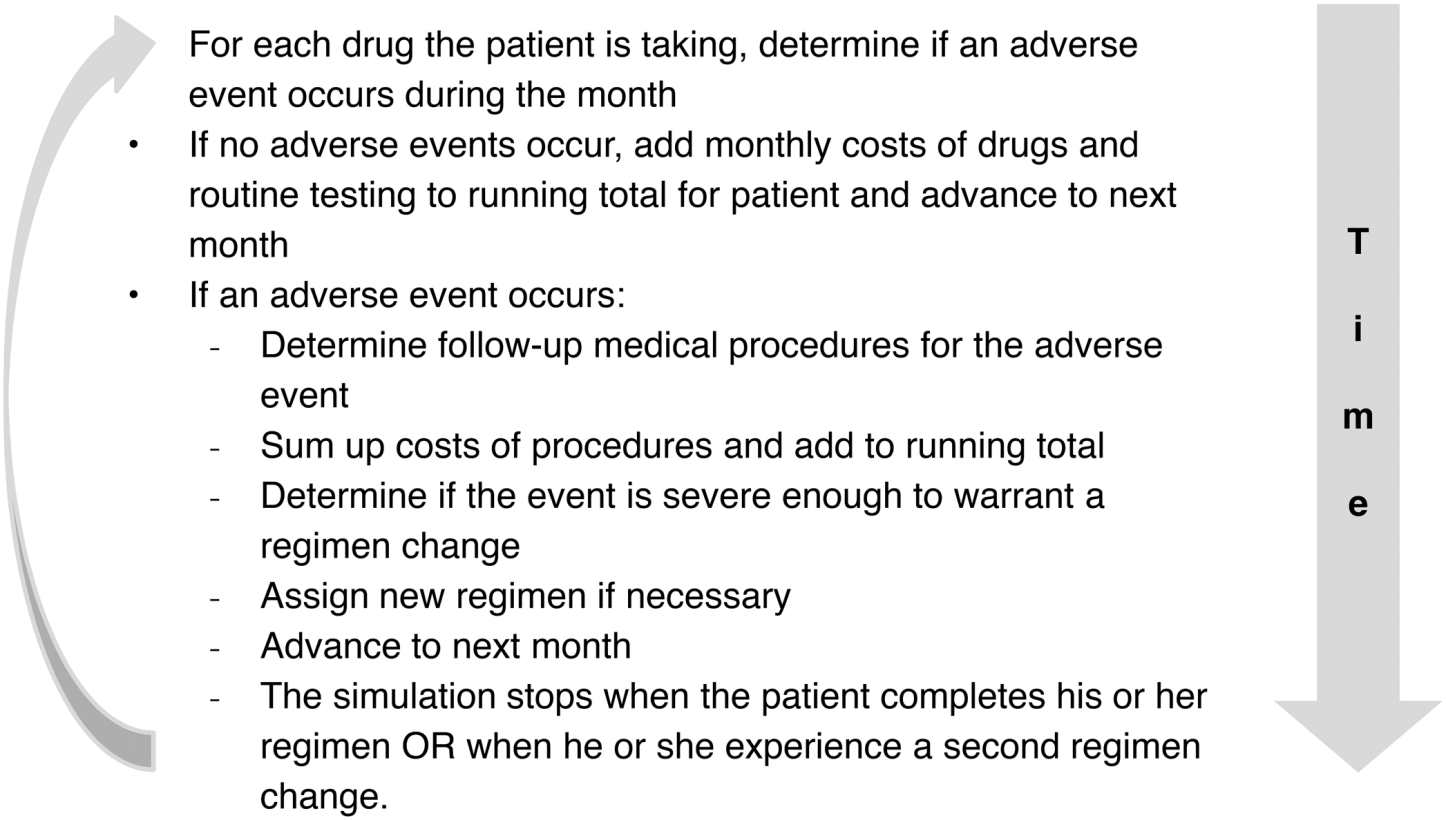
Actions simulated at each monthly time step.

We constructed a discrete-time stochastic model with a one-month time step for a hypothetical cohort of 100,000 TB patients. Fig 1 displays the actions performed during each monthly time step. Five initial TB regimens were considered, based on medication availability: 1) a standard regimen consisting of INH and RIF for six months supplemented with PZA and EMB for the first two months; 2) in the absence of INH, a regimen of RIF, PZA, and EMB for six months; 3) in the absence of RIF, a regimen of INH, EMB, and the second-line medication moxifloxacin (MFX) for 12 months supplemented with PZA for the first two months; 4) in the absence of both INH and RIF, a regimen of EMB, PZA, and MFX for 12 months supplemented with amikacin, another second-line medication, for the first six months; and 5) in the absence of PZA, a regimen of INH and RIF for nine months supplemented with EMB for the first two months (Table 1). Regimens were based on guidelines, clinical trials, and consultations with TB clinicians[7, 8]. Simulations were conducted using R 3.0 [9] (see S2 File for sample code).

**Table 1:** Regimens for the treatment of tuberculosis in a hypothetical cohort of TB patients in the setting of anti-TB drug shortages^a^ ^,^ ^b^.

Drug	Duration
Standard Therapy	
INH	6 mo
RIF	6 mo
EMB	2 mo
PZA	2 mo
No INH	
RIF	6 mo
EMB	6 mo
PZA	6 mo
No RIF	
INH	12 mo
EMB	12 mo
MFX	12 mo
PZA	2 mo
No PZA	
INH	9 mo
RIF	9 mo
EMB	2 mo
No INH or RIF	
PZA	18 mo
EMB	18 mo
MFX	18 mo
AMK	6 mo

^a^. Treatment regimens were based on literature and consultations with TB treatment experts. [8, 10–14]

^b^. Drug abbreviations: INH—isoniazid, RIF—rifampin, PZA—pyrazinamide, EMB—ethambutol, MFX—moxifloxacin, AMK—amikacin

In the cohort for our simulation, we assumed all patients had pan-sensitive pulmonary tuberculosis at treatment initiation. All 100,000 patients were placed on the same regimen and followed for the duration of therapy. This process was repeated for each of the five primary regimens At each one-month time step, occurrences of medication adverse events were calculated from published probabilities (Table 2). Cumulative incidences reported in the literature were converted to per-month probabilities using the formula *P*_*m*_ *= 1 − (1−P*_*c*_ *)* ^*t*^ where *P*_*m*_ is the per-month probability of the adverse event, *P*_*c*_ is the cumulative probability of the event, and *t* is duration of therapy in months. We allowed for mild, moderate, or severe adverse events for each medication. Mild events were those that cause slight discomfort or symptoms that would generally be tolerated. In practice, such an event might trigger a regimen change. However, for simplification and conservative cost estimates, we allowed regimen changes for only the proportion of cases involving mild vision loss or any moderate or severe event. Moderate events were defined as those that would require hospitalization or cause considerable discomfort, but were not immediately life-threatening. Events that would lead to extreme measures (e.g., liver transplantation) or were life-threatening were classified as severe. Patients experiencing a moderate or severe adverse event during the simulation were switched to an alternate regimen that excluded the offending medication. Based on the adverse event and the initial regimen, a patient was switched to a new regimen. Alternative regimens were developed in consultation with experienced TB physicians and based on published literature (see Tables A-D in S1 File).

**Table 2:** Probability and ranges of a medication adverse event.^a^ ^,^ ^b^

Adverse event and medication	Base probability	Low	High	Reference
Mild Hepatitis				
INH	0.006000	0.002000	0.018000	[15]
RIF	0.001500	0.000500	0.004500	[16, 17]
PZA	0.026400	0.008800	0.079200	[15]
PAS	0.005000	0.001667	0.015000	[18]
Moderate Hepatitis				
INH	0.000150	0.000050	0.000450	[15]
RIF	0.000038	0.000013	0.000113	assumed
PZA	0.003000	0.001000	0.009000	[15]
PAS	0.000500	0.000167	0.001500	assumed
Severe Hepatitis				
INH	0.000040	0.000013	0.000120	[15]
RIF	0.000010	0.000003	0.000030	assumed
PZA	0.000900	0.000300	0.002700	[15]
PAS	0.000050	0.000017	0.000150	assumed
Mild Vision				
EMB	0.002300	0.000767	0.006900	[19]
LNZ	0.132000	0.044000	0.396000	[20]
Moderate Vision				
EMB	0.000230	0.000077	0.000690	assumed
LNZ	0.013200	0.004400	0.039600	assumed
AMK nephrotoxicity				
Mild	0.150000	0.050000	0.450000	[21]
Moderate	0.015000	0.005000	0.045000	assumed
Severe	0.001500	0.000500	0.004500	assumed
AMK hearing loss				
Mild	0.370000	0.123333	0.500000	[21]
Moderate	0.037000	0.012333	0.111000	assumed
MFX tendinopathy				
Mild	0.001800	0.000600	0.005400	[22]
Moderate	0.000180	0.000060	0.000540	assumed
LNZ neuropathy				
Mild	0.325000	0.108333	0.500000	[23]
Moderate	0.032500	0.010833	0.097500	assumed
Severe	0.003250	0.001083	0.009750	assumed
LNZ myelosuppresion				
Mild	0.257000	0.085667	0.500000	[23]
Moderate	0.025700	0.008567	0.077100	assumed
Severe	0.002570	0.000857	0.007710	assumed
PAS gastrointestinal symptoms				
Mild	0.211000	0.070333	0.500000	[24]
Moderate	0.021100	0.007033	0.063300	assumed
Mild PAS hypothyroid	0.343000	0.114333	0.500000	[25]
CS central nervous system symptoms				
Mild	0.011000	0.003667	0.033000	[26]
Moderate	0.001100	0.000367	0.003300	assumed
Mild other adverse events				
INH	0.027000	0.009000	0.081000	[16, 27]
RIF	0.002600	0.000867	0.007800	[28]
PZA	0.400000	0.133333	0.500000	[13]
AMK	0.090000	0.030000	0.270000	[29]
MFX	0.251000	0.083667	0.500000	[30]
Moderate other adverse events				
INH	0.002700	0.000900	0.008100	assumed
RIF	0.000260	0.000087	0.000780	assumed
PZA	0.040000	0.013333	0.120000	assumed
AMK—pyr	0.009000	0.003000	0.027000	assumed
MFX	0.027000	0.009000	0.081000	[30]
MFX severe other adverse events	0.003000	0.001000	0.009000	[30]

^a^. Drug abbreviations: INH—isoniazid, RIF—rifampin, PZA—pyrazinamide, EMB—ethambutol, MFX—moxifloxicin, AMK—amikacin, CS—cycloserine, LNZ—linezolid, PAS—para-aminosalicylic acid

^b^. When literature was unavailable for moderate and severe adverse events, we followed a convention others have used and assumed the probabilities of these events were 1/10 and 1/100 the value of the mild event. For example, see [17]

Total treatment cost was computed by summing the costs incurred for medications, routine testing for medication toxicity monitoring, and management of adverse events based on 2013 prices (Table 3). We assumed that prices remained stable during the simulation throughout an individual’s treatment course. We consulted TB physicians to determine the routine tests for specific TB medications (see Tables A-D in S1 File) and the follow-up procedures for specific adverse events (see Tables A-D in S1 File). Cost estimates were based on current procedural terminology (CPT) codes or the literature [31]. The American Medical Association assigns a CPT code to every task and service a medical practitioner may provide to a patient which insurers then use to determine the reimbursement per service [31]. In some cases, 95% confidence intervals for adverse event probabilities were available (Table 2); however, the amount of uncertainty determined in an individual study is probably an underestimate of the true variation. For example, studies, sub-population risk factors, and medication duration and dosing could vary. Therefore, for our sensitivity analysis we calculated more conservative upper and lower bounds that were larger than published 95% confidence intervals by increasing and decreasing the base adverse event probability by a factor of three. To obtain conservative upper and lower bounds for each cost input, we increased and decreased our base cost by 30%. Since we were most interested in average regimen costs relative to the standard regimen, we computed cost ratios specific to each regimen using the average cost of standard therapy as the denominator. Upper and lower bounds for the proportion of patients experiencing at least one adverse event and the proportion of patients experiencing a regimen change were obtained by running the simulation with the upper and lower bounds for adverse event probabilities. Upper and lower bounds for cost ratios were obtained similarly. To determine the impact of increased medication costs on the average cost of treatment per patient, we ran simulations based on market price increases for first-line anti-TB medications from 2013 through 2014 as reported by TB controllers to NTCA. Cost escalations were 35, two, and three times higher than published costs for INH, RIF and PZA, respectively. Because we found multiple reports of cost increases for PZA, we used the lowest escalation to ensure the most conservative calculations [4, 6].

**Table 3:** Costs and ranges of medications, procedures and medical tests due to medication adverse event in US dollars, 2014.

Medication ^a^ ^,^ ^b^	Base Monthly Price	Low^c^	High^c^
INH	1.00	0.70	1.30
RIF	26.00	18.20	33.80
PZA	35.00	24.50	45.50
EMB	20.00	14.00	26.00
AMK	176.00	123.20	228.80
MFX	80.00	56.00	104.00
PAS	173.00	121.10	224.90
CS	435.00	304.50	565.50
LNZ	1064.00	744.80	1383.20
Procedures	Base Cost	Low	High
Acute hepatitis panel	65.47	45.83	85.11
Ankle magnetic resonance imaging (MRI)	547.77	383.44	712.10
Ankle radiograph	34.70	24.29	45.11
Arthrocentesis	45.93	32.15	59.71
Audiogram	42.88	30.02	55.74
Assay of calcium	7.09	4.96	9.22
Assay of creatinine	7.04	4.93	9.15
Assay of magnesium	9.21	6.45	11.97
Blood transfusion	34.70	24.29	45.11
Color vision examination	52.42	36.69	68.15
Comprehensive metabolic panel	14.53	10.17	18.89
Complete blood count	10.69	7.48	13.90
Electrolyte panel	9.64	6.75	12.53
Eye exam for a new patient	100.03	70.02	130.04
Hemodialysis	71.11	49.78	92.44
Hepatic function panel	11.23	7.86	14.60
Liver transplantation ^d^	230800.00	161560.00	300040.00
Nonsteroidal anti-inflammatory drugs	10.00	7.00	13.00
Office consultation (nephrology)	107.85	75.50	140.21
Office consultation (rheumatology)	107.85	75.50	140.21
Office consultation (surgery)	107.85	75.50	140.21
Office or outpatient visit for established patient (physician)	107.85	75.50	140.21
Office or outpatient visit for established patient (nurse)	8.85	6.20	11.51
One percent hydrocortisone cream	10.00	7.00	13.00
Physical therapy evaluation	31.98	22.39	41.57
Routine ECG with at least 12 leads; with interpretation and report	18.37	12.86	23.88
TB inpatient hospital day ^e^	1813.82	1269.67	2357.97
Therapeutic drug monitoring	619.00	433.30	804.70
Thyroid function panel	47.96	33.57	62.35
Treatment for gout with allopurinol and colchicine	20.00	14.00	26.00
Uric acid	6.21	4.35	8.07
Visual acuity screen	2.35	1.65	3.06

Sources: [10] and [31], except for Transplant, [32]

^a^. Medications were based on public health pricing and standard dosing: INH 300mg daily, RIF 600mg daily, PZA 25mg/kg daily, EMB 15mg/kg daily, Amikacin 15mg/kg daily, Moxifloxacin 400mg daily, PAS 6gm daily, Cycloserine 500mg daily, Linezolid 600mg daily.

^b^. Drug abbreviations: INH—isoniazid, RIF—rifampin, PZA—pyrazinamide, EMB—ethambutol, MFX—moxifloxacin, AMK—amikacin, CS—cycloserine, LNZ—linezolid, PAS—para-aminosalicylic acid

^c^. Reported ranges for costs are 30% lower and higher than the baseline costs and are the values used in simulations.

^d^. 40% of the billed cost of a liver transplantation as reported in the referenced source.

^e^. Adapted from [33]

No ethical human subjects review approval was required for this study because we gathered data from the literature and no patient-level data were accessed.

## Results

Cost ratios of the four candidate regimens utilized based on medication availability relative to the standard regimen are presented in Table 4. The cost ratio was 1.7 (1.2, 2.3) in the absence of INH, and 4.9 (3.2, 7.3) times higher in the absence of RIF. When both INH and RIF were absent, the cost ratio was 18.6 (10.0, 39.0). Average treatment cost in the absence of PZA was similar to the average treatment cost for standard treatment: cost ratio = 1.1 (0.7, 1.7). When prices for INH, RIF, and PZA were inflated, the average treatment cost for standard treatment increased by a factor of 2.7 (1.9, 3.0).

**Table 4:** Cost ratios of candidate regimens relative to the standard TB regimen.

Regimen^a^ ^,^ ^b^	Baseline Cost	Low	High	Baseline Plus^c^	Low Plus^c^	High Plus^c^
Standard	Ref: 1.00	0.68	1.51	2.74	1.90	3.00
No INH	1.71	1.18	2.25	3.71	2.59	3.87
No RIF	4.90	3.22	7.28	6.81	4.56	9.11
No PZA	1.08	0.72	1.71	2.96	2.03	3.33
No INH or RIF	18.61	10.03	38.95	22.99	13.10	42.67

^a^. Drug abbreviations: INH—isoniazid, RIF—rifampin, PZA—pryazinamide, EMB—ethambutol

^b^. Refer to Tables A-D in S1 File for regimens

^c^.”Plus” reflects added costs due to observed increased prices of INH (x35), pyrazinamide (x3), and rifampin (x2)

The percentage of patients experiencing at least one adverse event while receiving standard therapy was 3.9% (1.3%, 11.8%, Table 5). When RIF was unavailable, 51.5% (20.1%, 83.8%) of patients experienced at least one adverse event. In the absence of PZA 6.0% (2.1%, 17.2%) of patients experienced at least one adverse event. Only 0.7% (0.2%, 2.1%) of patients experienced an adverse event while receiving the “No INH” regimen (see Table 5). When both INH and RIF were unavailable, 82.5% (41.2%, 98.5%) of patients experienced at least one adverse event. Among patients who were receiving standard treatment, 0.3% (0.1%, 1.0%) experienced a regimen change that was in response to a moderate or severe adverse event. In the absence of RIF, 6.4% (2.3%, 18.3%) of patients experienced a regimen change. When only INH was unavailable, 0.2% (0.1%, 07%) of patients experienced a regimen change. When only PZA was unavailable 0.5% (0.2%, 1.5%) of patients experienced a regimen change. In the absence of both INH and RIF, 11.3% (4.0%, 30.4%) of patients experienced a regimen change.

**Table 5:** Proportion of patients projected to experience at least one adverse event, and proportion of patients projected to experience a regimen change, by initial regimen ^a^ ^,^ ^b^

Regimen	% with ≥1 adverse event	Low %	High %	% with a regimen change	Low %	High %
Standard	3.9	1.3	11.8	0.3	0.1	1.0
No INH	0.7	0.2	2.1	0.2	0.1	0.7
No RIF	51.5	20.1	83.8	6.4	2.3	18.3
No PZA	6.0	2.1	17.2	0.5	0.2	1.5
No INH or RIF	82.5	41.2	98.5	11.3	4.0	30.4

^a^. Drug abbreviations: INH—isoniazid, RIF—rifampin, PZA—pryazinamide, EMB—ethambutol

^b^. Refer to Tables A-D in S1 File for regimens

## Discussion

Interruptions in the supply of anti-TB medications are costly and can result in adverse events for patients; in the stochastic model constructed here, the cost ratio of TB treatment without INH, RIF, or PZA to standard treatment was 1.7, 4.9, and 1.1 times higher, respectively. Without both INH and RIF, the cost ratio was 18.6 times higher. Our conservative model illustrates the economic and health impacts that these interruptions and price escalations can have on both TB patients and health systems. Not only are alternative medication regimens more expensive in most instances, but patients are exposed to more toxic and less effective medications for longer durations of therapy. For example, in the absence of RIF, the most potent first-line medication, 51.5% of individuals are projected to experience an adverse event. This is likely attributable to patients needing 12 months, instead of 6 months with RIF, of treatment with INH and MFX which have higher rates of adverse events including INH-related hepatotoxicity based on published literature (Table 2).

Surveys conducted by the NTCA show that shortages have resulted in delays and interruptions in anti-TB treatment initiation, which could lead to increased TB transmission, and prioritization of patients to receive latent TB infection treatment, creating a missed opportunity to prevent future TB cases as asymptomatic individuals are unlikely to return for treatment initiation at a later date [3, 5]. Further, drug interruptions requiring regimens with AMK or LNZ can lead to long-term sequelae of hearing loss or peripheral neuropathy requiring lifelong care. These findings and results from our study highlight the need for ensuring a continuous and affordable supply of anti-TB medications.

Our study was subject to several limitations. While probabilities and costs in our model were based on the literature, in many instances, the data for our model parameters did not exist. In the absence of reports in the literature, we calculated conservative imputations (i.e. probabilities and costs biased towards zero). We did not include treatment efficacy in our model because our goal was to estimate incremental costs associated with medication shortages and escalating prices, and because randomized clinical trial efficacy data for alternative regimens, especially using second-line medications such as Linezolid, are sparse. Furthermore, as we did not account for additional costs resulting from TB morbidity and mortality, secondary TB cases, or administrative and overhead costs, our model represents a conservative cost estimate. Lastly, although several regimens can be substituted in the absence of INH and RIF, we focused on the most likely alternatives based on consultations with experts and the literature.

## Conclusion

Prompt diagnosis and initiation of treatment are the cornerstones to effective TB control. The 2012 INH shortage was unexpected and continued longer than forecasted, forcing TB programs nationally to quickly adapt and change treatment policies. Unfortunately, shortages in anti-TB medications have become more common in the last decade due to multiple factors including an inadequate supply of raw materials, manufacturing problems, and fewer manufacturers of anti-TB medications [3–5]. Over the last several years, public health programs have faced the predicament of how to maintain TB control with a limited supply of medications. Drug shortages affect individual patients, communities, hospitals, and public health programs. The NTCA has partnered with CDC and the Federal Drug Administration to build long-term solutions to ensure an adequate drug supply. We must not lose ground in our efforts towards TB elimination because of interruptions in anti-TB medications.

## Supporting Information

S1 File Supporting Tables for *Cost resulting from anti-tuberculosis drug shortages in the United States*: *a hypothetical cohort study*. This supplement provides additional details of the model structure and calculation of model inputs and includes the following tables:Table A in S1 File. Alternative regimens a patient could receive in the setting of a medication adverse event, by regimen number. Table B in S1 File. Alternative regimens a patient could receive in the setting of a medication adverse event, by adverse event. Table C in S1 File. Medical tests for routine medication monitoring. Table D in S1 File. Procedures and frequency of procedure after adverse events.
(DOCX)

S2 File File for *Cost resulting from anti-tuberculosis drug shortages in the United States*: *a hypothetical cohort study*. Example Rnw file that was used to simulate outcomes.
(RNW)
