# Correction: Cost Resulting from Anti-Tuberculosis Drug Shortages in the United States: A Hypothetical Cohort Study

**DOI:** 10.1371/journal.pone.0137812

**Published:** 2015-09-02

**Authors:** James C. Scott, Neha Shah, Travis Porco, Jennifer Flood

The Funding section is incorrect. The complete, correct Funding statement is: TCP acknowledges support of the MIDAS study NIH NIGMS U01-GM087728. The funders had no role in study design, data collection and analysis, decision to publish, or preparation of the manuscript.
