# Supplementary material for: Cost Resulting from Anti-Tuberculosis Drug Shortages in the United States: A Hypothetical Cohort Study
Source: PLoS One. 2015 Aug 18;10(8):e0134597. doi: 10.1371/journal.pone.0134597 (PMC4540488; doi:10.1371/journal.pone.0134597)
Supplement: S1 File — This supplement provides additional details of the model structure and calculation of model inputs and includes the following tables:Table A in S1 File. Alternative regimens a patient could receive in the setting of a medication adverse event, by regimen number. Table B in S1 File. Alternative regimens a patient could receive in the setting of a medication adverse event, by adverse event. Table C in S1 File. Medical tests for routine medication monitoring. Table D in S1 File. Procedures and frequency of procedure after adverse events. (DOCX) [file pone.0134597.s001.docx]

**S1 Supporting Tables for** ***Cost resulting from anti-tuberculosis drug shortages in the United States: a hypothetical cohort study.***

This supplement provides additional details of the model structure and calculation of model inputs and includes the following tables:

Table A. Alternative regimens a patient could receive in the setting of a medication adverse event, by regimen number

Table B. Alternative regimens a patient could receive in the setting of a medication adverse event, by adverse event

Table C. Medical tests for routine medication monitoring

Table D. Procedures and frequency of procedure after adverse events

**Table A. Alternative regimens a patient in the hypothetical cohort could be changed to in the setting of a medication adverse event.^a^**

| **Regimen** | **Medications** ^b^ | **Duration medication given (months)** |
| --- | --- | --- |
| 1 | INH  RIF  EMB  PZA | 6  6  2  2 |
| 2 | RIF  EMB  PZA | 6  6  6 |
| 3 | INH  EMB  MFX  PZA | 12  12  12  2 |
| 4 | EMB  PZA  MFX  AMK | 18  18  18  6 |
| 5 | INH  RIF | 9  9 |
| 6 | RIF  EMB  MFX | 12  12  2 |
| 7 | INH  PZA  EMB | 9  9  9 |
| 8 | INH  RIF  PZA | 6  6  2 |
| 9 | RIF  EMB  MFX | 9  9  9 |
| 10 | EMB  MFX  AMK  CS | 18  18  6  6 |
| 11 | RIF  PZA  MFX | 9  9  9 |
| 12 | INH  EMB  MFX  AMK | 18  18  18  2 |
| 13 | INH  MFX  PZA | 12  12  2 |
| 14 | INH  PZA  MFX  AMK | 18  18  18  2 |
| 15 | INH  EMB  PZA | 18  18  2 |
| 16 | EMB  MFX  CS  AMK | 18  18  18  6 |
| 17 | PZA  MFX  CS  AMK | 18  18  18  6 |
| 18 | PZA  EMB  CS  LNZ  AMK | 18  18  18  18  6 |
| 19 | PZA  EMB  MFX  LNZ  PAS | 18  18  18  18  18 |
| 20 | EMB  PZA  AMK  PAS | 18  18  6  18 |
| 21 | PZA  EMB  MFX  LNZ  CS | 18  18  18  18  18 |
| 22 | PZA  MFX  AMK | 18  18  6 |
| 23 | RIF  INH  EMB | 9  9  2 |
| 1. Treatment regimens were based on literature and consultations with TB treatment experts. [[1-11](#_ENREF_1)] 2. Drug abbreviations: INH - isoniazid, RIF - rifampin, PZA - pryazinamide, EMB - ethambutol, MFX - moxifloxacin, AMK - amikacin, CS - cycloserine, LNZ - linezolid, PAS – para-aminosalicylic acid | | |

**Table B. Alternative treatment regimens a patient can be changed to change due to an adverse event**

| **Medication-related adverse event** | | **New regimen and duration for each drug**^a, b^ |
| --- | --- | --- |
| Initial Regimen: Standard Therapy | | |
| INH | |  |
| Moderate Hepatitis | | RIF 6 - EMB 6 - PZA 6 |
| Severe Hepatitis | | RIF 12 - EMB 12 - MFX 2 |
| Moderate other event | | RIF 6 - EMB 6 - PZA 6 |
| RIF | |  |
| Moderate Hepatitis | | INH 12 - EMB 12 - MFX 12 - PZA 2 |
| Severe Hepatitis | | EMB 18 - MFX 18 - CS 18 - AMK 6 |
| Moderate other event | | INH 9 - PZA 9 - EMB 9 |
| PZA | |  |
| Moderate Hepatitis | | INH 9 - RIF 9 |
| Severe Hepatitis | | RIF 12 - EMB 12 - MFX 2 |
| Moderate other event | | INH 9 - RIF 9 |
| EMB  Vision impairment (any) | | INH 6 - RIF 6 - PZA 2 |
|  | |  |
| Initial Regimen: No INH regimen | | |
| RIF | |  |
| Moderate or hepatitis | | PZA 18 - EMB 18 - MFX 18 - AMK 6 |
| Severe Hepatitis | | EMB 18 - MFX 18 - CS 6 - AMK 6 |
| Moderate other event | | PZA 18 - EMB 18 - MFX 18 - AMK 6 |
| PZA  Adverse event (any) | | RIF 9 - EMB 9 - MFX 9 |
| EMB  Vision impairment (any) | | PZA 9 - RIF 9 - MFX 9 |
|  | |  |
| Initial Regimen: No RIF regimen | | |
| INH  Adverse event (any) | | PZA 18 - EMB 18 - MFX 18 - AMK 6 |
| PZA  Adverse event (any) | | INH 18 - EMB 18 - MFX 18 - AMK 2 |
| EMB | |  |
| Mild vision impairment | | INH 12 - MFX 12 - PZA 2 |
| Moderate vision impairment | | INH 18 - PZA 18 - MFX 18 - AMK 2 |
| MFX  Adverse event (any) | | INH 18 - EMB 18 - PZA 2 |
|  | |  |
| Initial Regimen: No INH or RIF regimen | | |
| PZA |  | |
| Hepatitis – moderate or severe | EMB 18 - MFX 18 - CS 18 - AMK 6 | |
| Moderate other event | EMB 18 - PZA 18 - AMK 6 - PAS 18 | |
| EMB |  | |
| Mild vision impairment | PZA 18 - MFX 18 - AMK 6 | |
| Moderate vision impairment | PZA 18 - MFX 18 - CS 18 - AMK 6 | |
| MFX  Adverse event (any) | PZA 18 - EMB 18 - LNZ 18 - CS 18 - AMK 6 | |
| AMK  Adverse event (any) | PZA 18 - EMB 18 - MFX 18 - LNZ 18 - PAS 18 | |
|  |  | |
| Initial regimen: no PZA regimen | | |
| INH  Any adverse event | RIF 9 - EMB 9 - MFX 9 | |
| RIF |  | |
| Hepatitis – moderate or severe | INH 18 - EMB 18 - MFX 18 - AMK 2 | |
| Severe Hepatitis | EMB 18 - MFX 18 - CS 18 - AMK 6 | |
| Moderate other event | INH 18 - EMB 18 - MFX 18 - AMK 2 | |
| EMB  Vision impairment (any) | INH 6 - RIF 6 - PZA 2 | |
| 1. Treatment regimens were based on literature and consultations with TB treatment experts. [[1-11](#_ENREF_1)] 2. Drug abbreviations: INH - isoniazid, RIF - rifampin, PZA - pryazinamide, EMB - ethambutol, MFX - moxifloxacin, AMK - amikacin, CS - CS, LNZ - linezolid, PAS – para-aminosalicylic acid | | |

**Table C. Medical Tests Associated with Routine Monitoring of Medication Side Effects^a,b^**

| Drug Name | Medical Test | Frequency |
| --- | --- | --- |
| INH | Hepatic function panel | baseline |
| AMK | Assay of creatinine | monthly |
| AMK | Electrolyte panel | monthly |
| AMK | Assay of magnesium | monthly |
| AMK | Assay of calcium | monthly |
| RIF | Hepatic function panel | monthly |
| EMB | Visual acuity screen | monthly |
| EMB | Color vision examination | monthly |
| PZA | Hepatic function panel | monthly |
| PZA | Uric acid | monthly |
| PAS | Hepatic function panel | monthly |
| PAS | Thyroid function panel | baseline |
| LNZ | Complete blood count | baseline |
| LNZ | Complete blood count | monthly |
| 1. Treatment regimens were based on literature and consultations with TB treatment experts. [[1-3](#_ENREF_1), [5](#_ENREF_5), [6](#_ENREF_6), [8](#_ENREF_8), [12](#_ENREF_12), [13](#_ENREF_13)] 2. Drug abbreviations: INH - isoniazid, RIF - rifampin, PZA - pryazinamide, EMB - ethambutol, MFX - moxifloxacin, AMK - amikacin, CS - CS, LNZ - linezolid, PAS – para-aminosalicylic acid | | |

**Table D. Procedures and frequency of procedure conducted for drug-specific adverse events^a^**

| Adverse Event**^b^** | Procedure | | | Estimated Quantity |
| --- | --- | --- | --- | --- |
| **INH** | | | | |
| Mild hepatitis |  | | |  |
|  | Office or outpatient visit for established patient | | | 1 |
|  | Hepatic function panel | | | 2 |
|  | Office or outpatient visit for established patient (nurse) | | | 1 |
| Moderate hepatitis |  | | |  |
|  | Office or outpatient visit for established patient | | | 2 |
|  | Hepatic function panel | | | 3.5 |
|  | Acute hepatitis panel | | | 1 |
|  | Office or outpatient visit for established patient (nurse) | | | 1 |
| Severe hepatitis |  | | |  |
|  | Liver transplantation | | | 1 |
| Mild other |  | | |  |
|  | Office or outpatient visit for established patient | | | 2 |
|  | Complete blood count | | | 1 |
|  | Office or outpatient visit for established patient (nurse) | | | 1 |
|  | Electrolyte panel | | | 1 |
| Moderate other |  | | |  |
|  | Office or outpatient visit for established patient | | | 3.5 |
|  | Complete blood count | | | 1 |
|  | Office or outpatient visit for established patient (nurse) | | | 1 |
|  | Electrolyte panel | | | 1 |
|  |  | | |  |
| **RIF** | | | | |
| Mild hepatitis |  | | |  |
|  | Office or outpatient visit for established patient | | | 1 |
|  | Hepatic function panel | | | 2 |
|  | Office or outpatient visit for established patient (nurse) | | | 1 |
| Moderate hepatitis |  | | |  |
|  | Office or outpatient visit for established patient | | | 2 |
|  | Hepatic function panel | | | 3.5 |
|  | Acute hepatitis panel | | | 1 |
|  | Office or outpatient visit for established patient (nurse) | | | 1 |
| Severe hepatitis |  | | |  |
|  | Liver transplantation | | | 1 |
| Mild other |  | | |  |
|  | Office or outpatient visit for established patient | | | 2 |
|  | Hepatic function panel | | | 2 |
|  | Complete blood count | | | 1 |
|  | Office or outpatient visit for established patient (nurse) | | | 1 |
|  | Topical cream | | | 1 |
| Moderate other |  | | |  |
|  | Office or outpatient visit for established patient | | | 3.5 |
|  | Hepatic function panel | | | 3.5 |
|  | Complete blood count | | | 3.5 |
|  | Office or outpatient visit for established patient (nurse) | | | 1 |
|  | TB inpatient hospital day | | | 1 |
|  |  | | |  |
| **EMB** | | | | |
| Mild vision |  | | |  |
|  | Office or outpatient visit for established patient | | | 1 |
|  | Eye exam for a new patient | | | 1 |
|  | Office or outpatient visit for established patient (nurse) | | | 1 |
| Moderate vision |  | | |  |
|  | Office or outpatient visit for established patient | | | 2 |
|  | Eye exam for a new patient | | | 2 |
|  | Office or outpatient visit for established patient (nurse) | | | 1 |
|  |  | | |  |
| **PZA** | | | | |
| Mild hepatitis |  | | |  |
|  | Office or outpatient visit for established patient | | | 1 |
|  | Hepatic function panel | | | 2 |
|  | Office or outpatient visit for established patient (nurse) | | | 1 |
| Moderate hepatitis |  | | |  |
|  | Office or outpatient visit for established patient | | | 2 |
|  | Hepatic function panel | | | 3.5 |
|  | Acute hepatitis panel | | | 1 |
|  | Office or outpatient visit for established patient (nurse) | | | 1 |
| Severe hepatitis |  | | |  |
|  | Liver transplantation | | | 1 |
| Mild other |  | | |  |
|  | Office or outpatient visit for established patient | | | 1 |
|  | Hepatic function panel | | | 2 |
|  | Uric acid | | | 1 |
|  | Complete blood count | | | 1 |
|  | Office or outpatient visit for established patient (nurse) | | | 1 |
|  | Topical cream for rash | | | 1 |
| Moderate other |  | | |  |
|  | Office or outpatient visit for established patient | | | 2 |
|  | Hepatic function panel | | | 3.5 |
|  | Uric acid | | | 2 |
|  | Complete blood count | | | 1 |
|  | Office or outpatient visit for established patient (nurse) | | | 1 |
|  |  | | |  |
| **AMK** | | | | |
| Mild nephrotoxicity |  | | |  |
|  | Office or outpatient visit for established patient | | | 2 |
|  | Comprehensive metabolic panel | | | 2 |
|  | Office or outpatient visit for established patient (nurse) | | | 1 |
| Moderate nephrotoxicity |  | | |  |
|  | Office or outpatient visit for established patient | | | 3.5 |
|  | Comprehensive metabolic panel | | | 5.5 |
|  | Therapeutic drug monitoring | | | 1 |
|  | Office or outpatient visit for established patient (nurse) | | | 1 |
| Severe nephrotoxicity |  | | |  |
|  | Office or outpatient visit for established patient | | | 5.5 |
|  | Comprehensive metabolic panel | | | 7.5 |
|  | Office consultation (nephrology) | | | 1 |
|  | Hemodialysis | | | 90 |
|  | TB inpatient hospital day | | | 1 |
| Mild hearing |  | | |  |
|  | Office or outpatient visit for established patient | | | 2 |
|  | Audiogram | | | 1 |
|  | Office or outpatient visit for established patient (nurse) | | | 1 |
| Moderate hearing |  | | |  |
|  | Office or outpatient visit for established patient | | | 3.5 |
|  | Audiogram | | | 2 |
|  | Comprehensive metabolic panel | | | 1 |
|  | Office or outpatient visit for established patient (nurse) | | | 1 |
| Mild other |  | | |  |
|  | Office or outpatient visit for established patient | | | 2 |
|  | Audiogram | | | 1 |
|  | Office or outpatient visit for established patient (nurse) | | | 1 |
| Moderate other |  | | |  |
|  | Office or outpatient visit for established patient | | | 3.5 |
|  | Audiogram | | | 2 |
|  | Comprehensive metabolic panel | | | 1 |
|  | Office or outpatient visit for established patient (nurse) | | | 1 |
|  |  | | |  |
| **MFX** | | | | |
| Mild tendinopathy |  | | |  |
|  | Office or outpatient visit for established patient | | | 3.5 |
|  | Hepatic function panel | | | 1 |
|  | Comprehensive metabolic panel | | | 1 |
|  | Office or outpatient visit for established patient (nurse) | | | 1 |
|  | Prescription for non-steriodal anti-inflammatory medication | | | 1 |
| Moderate tendinopathy |  | | |  |
|  | Office or outpatient visit for established patient | | | 4.5 |
|  | Hepatic function panel | | | 1 |
|  | Aspirate joint | | | 1 |
|  | Comprehensive metabolic panel | | | 1 |
|  | Complete blood count | | | 1 |
|  | Uric acid | | | 1 |
|  | Office consultation (rheumatology) | | | 1 |
|  | Ankle MRI | | | 1 |
|  | Office or outpatient visit for established patient (nurse) | | | 1 |
|  | Physical therapy evaluation | | | 2 |
| Mild other |  | | |  |
|  | Office or outpatient visit for established patient | | | 1 |
|  | Hepatic function panel | | | 1 |
|  | Office or outpatient visit for established patient (nurse) | | | 1 |
| Moderate other |  | | |  |
|  | Office or outpatient visit for established patient | | | 2 |
|  | Hepatic function panel | | | 1 |
|  | Comprehensive metabolic panel | | | 1 |
|  | Ankle MRI | | | 1 |
|  | Office or outpatient visit for established patient (nurse) | | | 1 |
|  | Routine ECG with at least 12 leads; with interpretation and report | | | 1 |
| Severe other |  | | |  |
|  | Office or outpatient visit for established patient | | | 3.5 |
|  | Hepatic function panel | | | 1 |
|  | Electrolyte panel | | | 1 |
|  | Ankle Magnetic resonance imaging (MRI) | | | 1 |
|  | Ankle radiograph | | | 1 |
|  | Office or outpatient visit for established patient (nurse) | | | 1 |
|  | Routine ECG with at least 12 leads; with interpretation and report | | | 1 |
|  | Office consultation (surgery) | | | 0 |
|  | TB inpatient hospital day | | | 1 |
|  |  | | |  |
| **LNZ** | | | | |
| Mild neuropathy |  | | |  |
|  | Office or outpatient visit for established patient | | | 2 |
|  | Complete blood count | | | 1 |
|  | Office or outpatient visit for established patient (nurse) | | | 1 |
|  | Electrolyte panel | | | 1 |
| Moderate neuropathy |  | | |  |
|  | Office or outpatient visit for established patient | | | 3.5 |
|  | Complete blood count | | | 1 |
|  | Office or outpatient visit for established patient (nurse) | | | 1 |
|  | Electrolytes panel | | | 1 |
| Severe neuropathy |  | | |  |
|  | Office or outpatient visit for established patient | | | 3.5 |
|  | Complete blood count | | | 1 |
|  | Office or outpatient visit for established patient (nurse) | | | 1 |
|  | Electrolyte panel | | | 1 |
| Mild myelosuppresion |  | | |  |
|  | Office or outpatient visit for established patient | | | 2 |
|  | Complete blood count | | | 3.5 |
|  | Office or outpatient visit for established patient (nurse) | | | 1 |
|  | Electrolyte panel | | | 1 |
| Moderate myelosuppresion |  | | |  |
|  | Office or outpatient visit for established patient | | | 2 |
|  | Complete blood count | | | 4.5 |
|  | Office or outpatient visit for established patient (nurse) | | | 1 |
|  | Electrolyte panel | | | 1 |
| Severe myelosuppresion |  | | |  |
|  | Office or outpatient visit for established patient | | | 4 |
|  | Complete blood count | | | 6.5 |
|  | TB inpatient hospital day | | | 1 |
|  | Electrolyte panel | | | 1 |
| Mild vision |  | | |  |
|  | Office or outpatient visit for established patient | | | 1 |
|  | Eye exam for a new patient | | | 1 |
|  | Office or outpatient visit for established patient (nurse) | | | 1 |
| Moderate vision |  | | |  |
|  | Office or outpatient visit for established patient | | | 2 |
|  | Eye exam for a new patient | | | 2 |
|  | Office or outpatient visit for established patient (nurse) | | | 1 |
|  |  | | |  |
| **PAS** | | | | |
| Mild gastrointestinal symptoms | |  |  |  |
|  | Office or outpatient visit for established patient | | | 2 |
|  | Hepatic function panel | | | 2 |
|  | Office or outpatient visit for established patient (nurse) | | | 1 |
|  | Electrolyte panel | | | 2 |
| Moderate gastrointestinal symptoms | | |  |  |
|  | Office or outpatient visit for established patient | | | 2 |
|  | Hepatic function panel | | | 2 |
|  | Office or outpatient visit for established patient (nurse) | | | 1 |
|  | Electrolyte panel | | | 2 |
| Severe gastrointestinal symptoms | |  |  |  |
|  | Office or outpatient visit for established patient | | | 3.5 |
|  | Hepatic function panel | | | 2 |
|  | Office or outpatient visit for established patient (nurse) | | | 1 |
|  | TB inpatient hospital day | | | 1 |
|  | Electrolyte panel | | | 3.5 |
| Mild hypothyroidism |  |  |  |  |
|  | Office or outpatient visit for established patient | | | 1 |
|  | Office or outpatient visit for established patient (nurse) | | | 1 |
| Mild hepatitis |  |  |  |  |
|  | Office or outpatient visit for established patient | | | 1 |
|  | Hepatic function panel | | | 2 |
|  | Office or outpatient visit for established patient (nurse) | | | 1 |
| Moderate hepatitis |  |  |  |  |
|  | Office or outpatient visit for established patient | | | 2 |
|  | Hepatic function panel | | | 3.5 |
|  | Acute hepatitis panel | | | 1 |
|  | Office or outpatient visit for established patient (nurse) | | | 1 |
| Severe hepatitis |  |  |  |  |
|  | Liver transplantation | | | 1 |
|  |  | | |  |
| **CS** | | | | |
| Mild central nervous system symptoms | | | | |
|  | Office or outpatient visit for established patient | | | 2 |
|  | Office or outpatient visit for established patient (nurse) | | | 1 |
|  | Therapeutic drug monitoring | | | 1 |
| Moderate central nervous system symptoms | | | | |
|  | Office or outpatient visit for established patient | | | 3.5 |
|  | Office or outpatient visit for established patient (nurse) | | | 1 |
|  | Therapeutic drug monitoring | | | 1 |
| 1. Treatment regimens were based on literature and consultations with TB treatment experts. [[1-3](#_ENREF_1), [5](#_ENREF_5), [6](#_ENREF_6), [8](#_ENREF_8), [12](#_ENREF_12), [13](#_ENREF_13)] 2. Drug abbreviations: INH - isoniazid, RIF - rifampin, PZA - pryazinamide, EMB - ethambutol, MFX - moxifloxacin, AMK - amikacin, CS - CS, LNZ - linezolid, PAS – para-aminosalicylic acid | | | | |

**References**

1. Centers for Disease Control and Prevention. Treatment of Tuberculosis, American Thoracic Society, CDC, and Infectious Diseases Society of America. MMWR. 2003;52(RR-11).

2. Curry International Tuberculosis Center. Tuberculosis Drug Information Guide. San Francisco: 2012.

3. Cattamanchi A, Dantes RB, Metcalfe JZ, Jarlsberg LG, Grinsdale J, Kawamura LM, et al. Clinical Characteristics and Treatment Outcomes of Patients with Isoniazid-Monoresistant Tuberculosis. Clinical Infectious Diseases. 2009;48(2):179-85. doi: 10.1086/595689.

4. Cox H, Ford N. Linezolid for the treatment of complicated drug-resistant tuberculosis: a systematic review and meta-analysis. Int J Tuberc Lung Dis. 2012;16(4):447-54. Epub 2012/02/14. doi: 10.5588/ijtld.11.0451

0451 [pii]. PubMed PMID: 22325685.

5. Hwang TJ, Wares DF, Jafarov A, Jakubowiak W, Nunn P, Keshavjee S. Safety of cycloserine and terizidone for the treatment of drug-resistant tuberculosis: a meta-analysis [Review article]. The International Journal of Tuberculosis and Lung Disease. 2013;17(10):1257-66. doi: 10.5588/ijtld.12.0863.

6. Steele M, Des Prez R. The Role of Pyrazinamide in Tuberculosis Chemotherapy. Chest. 1988;94(4):845-50.

7. Sotgiu G, Centis R, D'Ambrosio L, Alffenaar J, Anger H, Caminero J, et al. Efficacy, safety and tolerability of linezolid containing regimens in treating MDR-TB and XDR-TB: systematic review and meta-analysis. European Respiratory Journal. 2012;40(6):1430-42.

8. Fox W, Ellard GA, Mitchison DA. Studies on the treatment of tuberculosis undertaken by the British Medical Research Council Tuberculosis Units, 1946&#8211;1986, with relevant subsequent publications. The International Journal of Tuberculosis and Lung Disease. 1999;3(10):S231-S79.

9. Bastos ML, Hussain H, Weyer K, Garcia-Garcia L, Leimane V, Leung CC, et al. Treatment outcomes of patients with multidrug- and extensive drug-resistant tuberculosis according to drug susceptibility testing to first- and second-line drugs: an individual patient data meta-analysis. Clinical Infectious Diseases. 2014. doi: 10.1093/cid/ciu619.

10. Falzon D, Gandhi N, Migliori GB, Sotgiu G, Cox HS, Holtz TH, et al. Resistance to fluoroquinolones and second-line injectable drugs: impact on multidrug-resistant TB outcomes. European Respiratory Journal. 2013;42(1):156-68. doi: 10.1183/09031936.00134712.

11. Ahuja SD, Ashkin D, Avendano M, Banerjee R, Bauer M, Bayona JN, et al. Multidrug resistant pulmonary tuberculosis treatment regimens and patient outcomes: an individual patient data meta-analysis of 9,153 patients. PLoS Med. 2012;9(8):e1001300. Epub 2012 Aug 28.

12. Babu Swai O, Aluoch JA, Githui WA, Thiong'o R, Edwards EA, Darbyshire JH, et al. Controlled clinical trial of a regimen of two durations for the treatment of isoniazid resistant pulmonary tuberculosis. Tubercle. 1988;69(1):5-14. Epub 1988/03/01. PubMed PMID: 3051607.

13. Peloquin CA, Berning SE, Nitta AT, Simone PM, Goble M, Huitt GA, et al. Aminoglycoside Toxicity: Daily versus Thrice-Weekly Dosing for Treatment of Mycobacterial Diseases. Clinical Infectious Diseases. 2004;38(11):1538-44. doi: 10.1086/420742.
